# Refining a low-cost dermatophyte sampling method to enhance culture purity in resource-limited clinical settings in Bangladesh

**DOI:** 10.1016/j.mex.2026.103840

**Published:** 2026-02-20

**Authors:** Mahmudul Hasan, Shinjon Ahmed, Ezaz Mahmud Sabit, Shariful Islam Sobuz, Md. Abdullah Al Safi, Md. Toslim Mahmud, Firoz Ahmed, Sabuj Baran Dhar

**Affiliations:** a Department of Microbiology, Noakhali Science and Technology University, Noakhali-3814, Bangladesh; b Department of Electronics and Communication Engineering, Khulna University of Engineering & Technology, Khulna-9203, Bangladesh; c Department of Dermatology and Venereology, Noakhali Medical College, Noakhali, 3821, Bangladesh

**Keywords:** Dermatophytes, Mycological sampling methods, Low-cost laboratory technique, High-burden settings, Method optimization, Pure culture recovery

## Abstract

Dermatophytosis is an emerging public health concern in developing countries, with rising infection rates and persistent disease burden. Reliable isolation of dermatophytes is essential for effective treatment, surveillance, and downstream analyses, yet it remains challenging in low-resource clinical settings. We evaluated three mycological sampling approaches, including one conventional and two modified techniques to improve the recovery of pure dermatophytes while reducing contamination. The modified approaches comprised a heat-assisted aseptic sampling method to limit airborne contamination and a paper-zip transport approach using heat and sterile filter paper sealed in zip-lock bags before inoculation into the culture medium. The outcomes of all sampling approaches were subsequently assessed using conventional culture techniques. A total of 198 clinical specimens from 66 individuals generated 276 culture outcomes. Statistical analysis showed significant differences among methods in recovery of pure isolates and contamination control, with the Paper-Zip method exhibiting the highest selectivity (*p* < 0.05). Sensitivity analysis using conventional culture techniques showed similar diagnostic performance across the approaches, reflecting improvements in dermatophytes recovery.

Heat-assisted and Paper-Zip sampling improve recovery of pure dermatophytes while minimizing contamination.

Diagnostic sensitivity is consistent across all sampling approaches.

The study demonstrates practical, low-cost implementable options for routine diagnostics in resource-limited clinical settings.

## Graphical abstract

**Figure:**
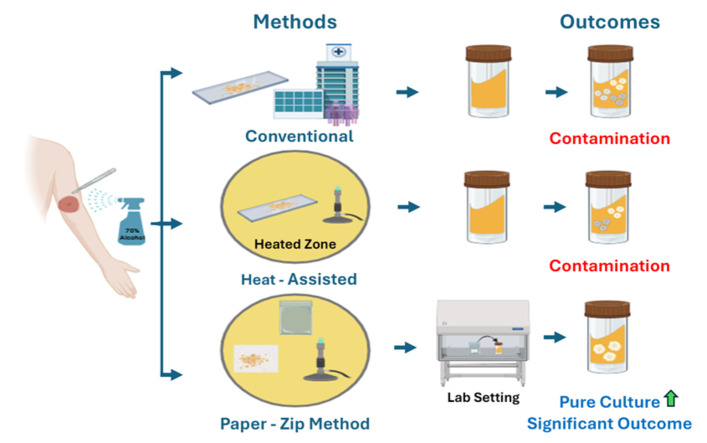

## Related research article

None

Specifications table

**Table:** 

Subject area	Immunology and Microbiology
**More specific subject area**	Medical Mycology; Dermatophyte Diagnostics; Low-Cost Clinical Microbiology Methods; Fungal Culture Optimization in Resource-Limited Settings
**Name of your method**	Low-Cost Enhanced Dermatophyte Sampling and Pre-Culture Debridement Method
**Name and reference of the original method**	The method refines the conventional dermatophyte sampling approach described in Weitzman & Summerbell (1995). “The dermatophytes.” Clin Microbiol Rev 8(2): 240–259.
**Resource availability**	Standard low-cost items: disposable scalpel blades, sterile Whatman filter paper, sterile zip lock bag, slide covers, 70 % ethanol, portable butane flame or spirit lamp, Sabouraud dextrose agar with chloramphenicol, gentamicin, cycloheximide, and Supplementary File −1.

## Background

Dermatophytosis is a superficial fungal infection that targets keratinized structures, including the skin, hair, and nails, in both humans and animals. It represents the most common category of fungal infections globally, comprising nine genera and over fifty species [1]. *Trichophyton, Microsporum*, and *Epidermophyton* represent the three major genera that infect humans, with particular species also including zoophilic varieties [2,3]. *Nannizia* species are predominantly geophilic but can also infect both animals and humans [2]. Dermatophytosis infects approximately 20–25 % of the global population, and accounts for a significant percentage of superficial mycoses and considerable morbidity and socioeconomic loss [4]. The prevalence is disproportionately higher in developing countries, and the incidence can reach as high as 40–60 % depending on geographic location, where determinants, including suboptimal hygiene, decreased access to healthcare services, and favorable climatic factors, promote easy transmission [5].

The tropical monsoon environment of Bangladesh, marked by high humidity, frequent rainfall, and elevated temperatures promotes the growth of dermatophytes, and creates optimal conditions for infection, reinfection, and prolonged disease duration [4]. Transmission is further facilitated by overcrowding, inadequate infection control, and low patient awareness. The country's abysmal doctor-to-patient ratio, with an average of 7.6 per 10,000 population, in comparison to WHO guidelines, further intensifies the issue [6]. The presence of inadequate laboratory infrastructure, a lack of qualified mycologists, and difficulties in specimen acquisition impede accurate diagnosis, leading to frequent misdiagnoses and suboptimal treatment outcomes. To address these issues, conventional diagnostic methods such as direct microscopic examination, biochemical assays, molecular assays, and the widely used culture method need to employ to properly isolate and characterize the pathogens [7].

Accurate sample collection is crucial for effective laboratory diagnosis, yet inadequate sampling remains a significant challenge in clinical practice. Traditional methods, including skin scrapings, pulled hairs, and nail clippings, are non-invasive but are associated with a 30 % rate of false-negative cultures [8]. Earlier research in Bangladesh also indicated a low culture-positive rate of 30.89 %, which highlights serious concerns regarding disease diagnosis [9]. This is often attributed to poor fungal recovery, contamination, or complications during transit, underscoring the need for improved methodologies in research to enhance diagnostic accuracy. Additionally, studies indicate that elevated population density leads to significant sources of fungal spores, attributed to the high levels of ambient particles [10], commonly present in Government Hospitals in Bangladesh.

In our research, we applied three distinct sampling approaches which incorporate commonly practiced pre-analytical steps such as alcohol disinfection alcohol, blade scraping, and preincubation before plating. Alternative techniques, adapted from the current method, emphasize alterations in pre-analytical factors such as disinfection protocols, transfer mediums, and transport conditions. The outcomes of these sampling approaches were evaluated using culture-based techniques. Yet, a universal consensus on the optimal balance between diagnostic sensitivity, contamination control, and logistical convenience remains elusive, especially in resource-limited settings like Bangladesh. Given the challenges of maintaining diagnostic sensitivity while minimizing contamination in routine clinical laboratories, this study aimed to systematically compare conventional and modified sampling approaches to optimize dermatophyte isolation in resource-limited settings in Bangladesh.

## Method details

A potential laboratory-based comparative study was conducted at Noakhali Science and Technology University from March 1, 2025, to May 30, 2025. The study protocol was reviewed and approved by the Ethics Clearance Committee of Noakhali Science and Technology University. Individuals were enrolled based on a clinical diagnosis of dermatophytosis, characterized by erythematous, scaling lesions with active margins and associated symptoms. Cases of ulceration or those presenting with secondary bacterial infections were excluded from the study. In addition, a written informed consent was obtained from all participants.

### Sample collection site description

Clinical samples were collected in a routine outpatient clinical room, sized approximately 7 × 7 feet, without air conditioning, and UV sterilization. Ventilation was provided by a window and a ceiling fan, which was switched off during the collection of the sample. The room had a tiled floor and was cleaned using standard dust-cleaning practices. Sample collection was conducted two days per week. On remaining days, the room was used for other clinical purposes. Approximately 50–70 individuals passed through the room on sample collection days. These conditions reflect routine diagnostic practices in government general hospitals in Bangladesh. During sample collection, only four people comprising one skilled employee for collecting samples, one associate, a patient and an attendant were allowed at a time. To reduce the possibility of cross-contamination, every instrument used was sterile and handled with care.

### Sample collection and transportation

The conventional dermatophyte sampling systems, particularly within crowded government hospitals and resource-limited settings like those in Bangladesh, often face high contamination by unwanted organisms and a loss of viable and recoverable pure isolates, which are common obstacles. To address these limitations, we modified the existing sample collection method and transportation process to attain greater purity and lower contamination in conventional culture at minimal cost. A total of 198 clinical specimens were obtained from 66 patients with active dermatophyte infections at the patient service delivery centre of the Department of Dermatology and Venerology, Noakhali Medical College (Geolocation: 22.87265°N, 91.08850°E). The attending microbiologist gathered samples following three distinct approaches from each individual (66 × 3 = 198), maintaining aseptic conditions.

### Method 1 (Conventional method)

Clinical samples were collected at a small collection area adjacent to the dermatology unit where patients were examined. In the conventional technique, the lesion periphery was sanitized with 70 % ethanol and allowed to air-dry for 30 s before scraping the active edge 5–10 times with a sterile surgical blade. Samples were collected on sterile glass slides and then put into pre-cooled (4 °C) Sabouraud Dextrose Agar with chloramphenicol vials supplemented with cycloheximide (125 µg/mL) and gentamicin (0.4 µg/mL) (SDCA+CG). These vials were then transported under refrigeration and incubated at 30 °C for 3 to 5 days, with daily checks for early development and purity. After preliminary growth was confirmed, the samples were sub-cultured onto SDCA+CG agar plates for 5 to 7 days to produce mature colonies and conduct additional testing.

### Method 2 (Heat-Assisted method)

In the second approach, we employed a controlled heat-assisted technique to reduce environmental contamination. A spirit lamp flame was used at a distance of 1–2 feet to establish a localized sterile zone around the sampling area, grounded in the hypothesis that this would minimize airborne spores and unwanted pathogens in the confined and densely populated hospital environment. Then, glass slides and blades were subjected to brief heating and cooling before use for specimen collection. Samples were collected according to **Method 1** and subsequently transferred from glass slides to sterile SDCA+CG vials, which were positioned within the flame zone. The following steps are identical to those outlined in **Method 1**.

### Method 3 (Paper-Zip method)

In the third and final approach, the same sampling procedure was adhered to as outlined in Method 2. The only operational adjustment involved was the post-collection handling; following the collection process, each specimen was placed onto autoclaved Whatman filter paper instead of a sterile glass slide, on the idea that filter paper's capacity to absorb moisture would inhibit the growth of mold and other undesirable organisms. The filter paper was subsequently folded and securely sealed in a zip-lock bag for transport to the laboratory, ensuring it was maintained at 4 °C or normal temperature. Upon receipt of the samples in the Mycology lab of the Department of Microbiology at NSTU, they are promptly placed onto SDCA+CG medium within the biosafety cabinet, followed by incubation and observation in accordance with established procedures.

### Post-incubation macroscopic observation and data collection

Following the incubation period, initial observations were documented via a macroscopic (naked eye) assessment of the vial before transferring it to a culture plate for further growth. The primary outcomes assessed included *Candida* spp., contamination (bacterial or mold overgrowth that obscures dermatophytes), dermatophytes (presence of dermatophyte growth in culture with concurrent growth of other fungi or contaminants), no growth (absence of fungal growth after five days), and pure dermatophytes (cultures in which dermatophytes were isolated exclusively) and documented appropriately.

### Statistical assessment of method performance and reliability

The statistical analysis was primarily conducted with SPSS version 26 (IBM) software, along with Power BI and Excel Power Query for data handling and visualization. To improve the quality and depth of the analysis, several Python libraries were integrated. Chi-square and Fisher's exact tests were used for categorical variables, such as growth rates and contamination. In contrast, ANOVA or Kruskal-Wallis tests were employed for time-to-positivity based on the data distribution. We assessed the sensitivity of various methods and subsequently employed the chi-square method to calculate the p-value, which aids in comprehending the significance of different factors. Additionally, we conducted Cramer’s V analysis to evaluate the strength of this significance, establishing a statistical significance level at a p-value of <0.05. The newly proposed method was clarified through the application of Fisher’s exact test.

### Quality control

To maintain quality control, laboratory technicians were kept uninformed about the collection method. Empty SDCA+CG vials were used as negative controls to prevent contamination of the media. Additionally, previously lab-grown isolates were cultivated in each batch to serve as a positive control. Aseptic conditions and sterile equipment were used correctly, and data were accurately documented by two different individuals, as this involved naked-eye observation. The optimal temperature during transportation and incubation was consistently maintained throughout the analysis. All analyses were conducted under blinded conditions, with sample identities revealed only at the final stage of data interpretation.

## Method validation

### Sample characteristics

**Fig. 1:**
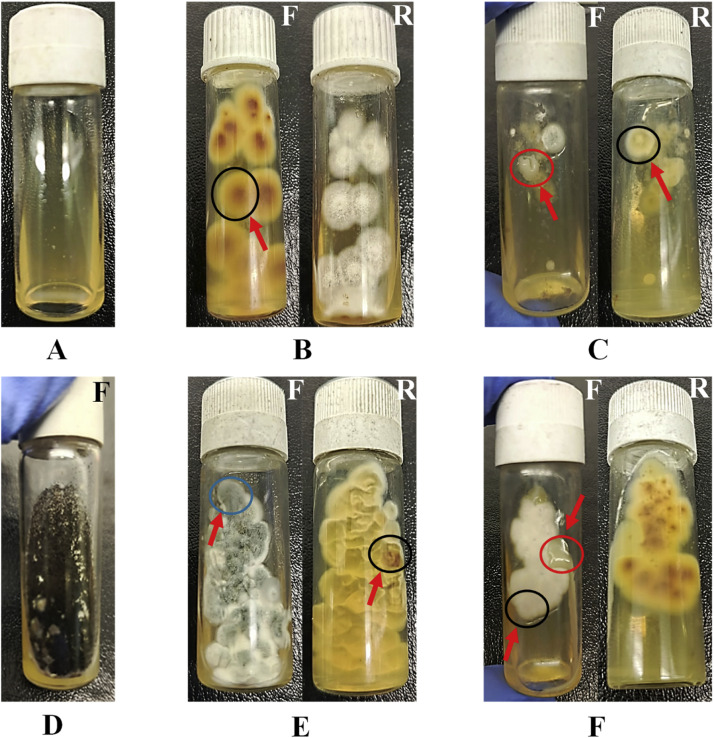
Distribution of culture outcomes from clinical samples is detailed in six panels (A-F). Panel A indicates no growth, Panel B reveals pure dermatophytes, Panel C represents mixed growth of dermatophytes (black circle) and Candida (red circle) contamination, Panel D shows all contamination, Panel E illustrates contamination in blue circle nearly obscuring dermatophytes. Panel F depicts both Candida spp. and dermatophytes together.

A total of 276 diagnostic-outcome events were recorded from 198 clinical specimens, utilizing three different diagnostic sampling methods. The results were categorized into various groups, including *Candida* spp., contamination, dermatophytes, no growth, and pure dermatophytes (keratinophilic fungi). In Fig. 1, Panels A through F consecutively display the real scenarios we encountered in the lab. The most prevalent contaminant is likely *Aspergillus* spp., which, due to its rapid growth, can overshadow the growth of dermatophytes (Fig. 1E). In contrast, dermatophytes grow more slowly, taking 5 to 7 days, making it nearly impossible to isolate pure dermatophytes from clinical specimens. This situation necessitates the modification to enhance the recovery of pure dermatophytes and reduce contamination. A total of 29.7 % (82 outcomes) were identified as pure dermatophytes among all clinical specimens (198), which served as the primary focus of this research.

### Distribution of culture outcomes across three sampling methods

**Fig. 2:**
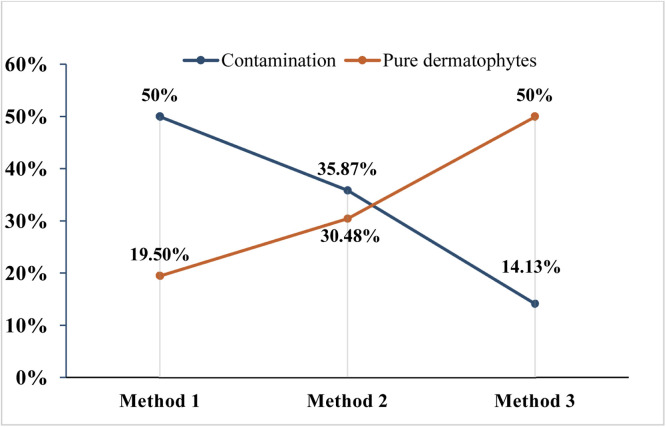
A line graph illustrating the increase in pure dermatophytes alongside the corresponding reduction in contamination rates, presented as percentages across various methods.

The conventional culture method was used to measure the outcome of the three different sampling approaches. A significant difference in the distribution of culture outcomes was observed across the methods presented in Table 1, while Table 2 illustrates the anticipated rate of these outcomes. Method 3 yielded the highest recovery of pure dermatophyte isolates, at 50 %, whereas the other methods showed recoveries ranging from 19 % to 31 %. The total number of pure dermatophytes in Method 3 is 41, which is significantly higher than the expected value of 22.87 (Table 2), indicating effective control of contamination. Method 3 significantly surpassed the recovery of pure dermatophytes using methods 2 and 1. Method 2 produced 25, slightly lower than its expected count of 27.3. Additionally, in Method 1, the value is 16, which is significantly lower than the expected 31.78. Method 1 showed a higher percentage of *Candida* spp. Contamination and a reduced recovery of pure dermatophytes (15.79 %) compared to its expected value of 31.78, reflecting lower selectivity. Method 3 achieved the lowest contamination rate at 14.13 %, while method 2 recorded a rate of 35.9 % and method 1 had a rate of 50 %. The lack of growth for method 3 also indicated a better isolation rate. It has been observed that methods that limit contamination percentage correspond with higher pure dermatophyte yields, highlighting an inverse relationship between pure dermatophyte yield and contamination. Overall, the results show that the methodological enhancements in the method 3 approach improved the purity and recovery of dermatophytes, while also minimizing unwanted microbial interference and reducing contamination (Fig. 2). The expected frequencies represent the distribution that would occur if no difference existed among the three diagnostic methods, calculated by following the equatison.

**Table 1:** The distribution of different outcomes varied substantially across methods.

Types	*Candida* spp.	Contamination	Dermatophytes ^a^	No growth	Pure Derma- tophytes^b^	Total	Proportion of pure dermatophytes
Method 1	10	46	30	5	16	107	19.51 %
Method 2	3	33	22	9	25	92	30.49 %
Method 3	3	13	8	12	41	77	50.00 %
Total	16	92	60	26	82	276	-

^a^Dermatophytes growth in culture with concurrent growth of other fungi or contaminants.

ᵇDermatophytes are isolated exclusively, without *Candida* spp., *Aspergillus* spp., other molds, or bacterial contamination.

**Table 2:** The expected frequencies of different culture outcomes across methods.

Method types	*Candida* spp.	Contamination	Dermatophytes	No-growth	Pure dermatophytes
Method 1	6.203	35.667	23.261	10.079	31.789
Method 2	5.333	30.667	20	8.667	27.333
Method 3	4.463	25.667	16.739	7.254	22.877

(1)

### Association between method and culture outcomes (Chi-square test)

The differences in culture outcomes among the three diagnostic methods were tested using χ² analysis to determine whether they were statistically significant or due to chance. The χ² analysis yielded a highly significant p-value (< 0.05), indicating that the diagnostic performances were notably different. Method 2 showed no significant deviations across any category (all *p* > 0.05), while method 1 demonstrated significance for pure dermatophyte recovery (*p* = 0.005) with a significant overall effect (*p* = 0.0014). Method 3 showed the strongest statistical performance, revealing substantial differences in contamination (*p* = 0.012), dermatophytes (*p* = 0.033), pure dermatophytes (*p* = 0.00015), and overall distribution (8.7 × 10⁻⁶). These results indicate that method 1 falls short in the key target category, while method 3 shows strong directional effects that improve pure dermatophyte recovery while minimizing contaminants Table 3.

**Table 3:** Chi-square significance levels for each outcome category by different methods.

Method types	*Candida* spp. (p)	Contamination (p)	Dermatophytes (p)	No growth (p)	Pure dermatophytes (p)	Overall p-value
Method 1	0.127	0.084	0.162	0.11	0.005	0.0014
Method 2	0.312	0.673	0.655	0.91	0.655	0.807
Method 3	0.488	0.012	0.033	0.078	0.00015	8.7 × 10⁻⁶

### Sensitivity analysis

Sensitivity analysis was conducted utilizing conventional culture to assess each method's ability to identify actual dermatophyte-positive specimens, including keratinophilic dermatophyte species correctly. Sensitivity represents the proportion of true positives correctly identified by the test and is calculated using the formula:

(2)

A true positive (TP) indicates a specimen that is correctly identified as dermatophyte positive. At the same time, a false negative (FN) occurs when a specimen containing dermatophytes is reported as negative or has no growth. Among the methods approached, method 3 had the highest sensitivity of 72.7 %, followed by method 1 at 71.2 %, and method 2 at 68.2 % (Table 4).

**Table 4:** Sensitivity of each method for dermatophytes culture.

Method types	True positive	False negative	Sensitivity ( %)
Method 1	47	19	71.2
Method 2	45	21	68.2
Method 3	48	18	72.7

### Influence of demographic and clinical factors

Chi-square tests were also conducted to analyze the relationship between patient-related factors and the results of fungal isolation. The results of χ² analyses showed that drug history (χ² = 11.72, *p* = 0.0196), sex (χ² = 9.53, *p* = 0.0491), and treatment modality (χ² = 22.38, *p* = 0.0043) were significantly related to the outcome of isolation (Supplementary Table S1). Thus, it appears that past antifungal use, gender-related biological differences, and treatment modality influenced the outcomes. Age range was not significantly related (χ² = 7.47, *p* = 0.825). From this observation, it is clear that both methodological and patient-related variables play a role in the success of dermatophyte culture, with some clinical factors potentially influencing the recovery rate.

### Pairwise comparisons for pure dermatophyte recovery (Fisher’s exact test & odds ratio)

An odds ratio (OR) analysis was performed to quantify the relative effectiveness of each sampling method in culturing the pure dermatophyte isolates. This statistical measure expresses the likelihood of a successful outcome compared with a reference method. This study found that method 3 resulted in a significantly higher isolation of pure dermatophytes (41 compared to 16) and was also utilized as the reference (OR = 1.0). Method 1 had an OR of 0.15, reflecting that it was 85 % less likely to culture pure dermatophytes compared to method 3 (Table 5). In comparison, method 2 presented an OR of 0.47, thus performing better than method 1 but still considerably less effectively than method 3. Odds ratio analysis was especially relevant here, as it provides a direct comparison of diagnostic strength across methods. The result shows that the changes made in method 3 significantly improve selective recovery performance in critical areas, reducing mixed growth and enhancing the isolation of true dermatophytes.

**Table 5:** Pairwise odds ratios with corresponding Fisher's exact test p-values showing the likelihood of contamination and pure dermatophytes recovery, highlighting relative performance differences among the three methods.

Category	Method 1 vs 2	Method 1 vs 3	Method 2 vs 1	Method 2 vs 3
Contamination	1.3482 (*p* = 0.3138)	3.7125 (*p* = 0.00021)	0.7417 (*p* = 0.3138)	2.7536 (*p* = 0.00883)
Pure Dermatophytes	0.4712 (*p* = 0.03655)	0.1544 (*p* < 0.00001)	2.1222 (*p* = 0.03655)	0.3276 (*p* = 0.00083)

Here, p-values indicate statistical significance from Fisher’s Exact Test: *p* < 0.05 is considered significant, and the odds ratio of 0.154<<1 indicates that method 1 decreases the chance of getting the pure dermatophytes, while method 3 highly increases the chance of getting the pure dermatophytes.

### Overall interpretation and methodological implications

We acknowledge that the collection conditions were not ideal and that environmental control was limited. However, these conditions reflect the true representation of clinical practice in government general hospitals in Bangladesh. The study therefore proposes and evaluates a sampling approach specifically designed to improve culture recovery under such real-world, resource-limited conditions. Overall, method 3 consistently outperformed methods 1 and 2 across all analyses, yielding the highest proportion of pure dermatophyte isolates while maintaining the lowest contamination rate and the highest sensitivity. The chi-square analysis showed that these differences are significant, and the odds ratio analysis highlighted the considerable benefit of method 3. The increased true-positive detection and lower rate of false negatives further support its diagnostic accuracy. Method 1, although traditionally used, showed the least effectiveness, resulting in low recovery rates and high contamination levels, which undermined its reliability for use in crowded settings. Method 2 showed some improvement, but it didn't have the same level of diagnostic strength as method 3. In method 2, no significant difference in contamination or yield was observed between the pre-heated and non-heated tools. These findings highlight method 3 as a dependable, sensitive, and statistically validated diagnostic sampling approach for the recovery of pure dermatophytes through culture-based outcome assessment. Furthermore, all the methods included here were cost-effective and utilized simple equipment and supplies. Method 3 has been demonstrated to be a practical approach for obtaining consistent, positive, and pure cultures outcomes considering its utilization of basic equipment’s and minimal cost for resource limited clinical settings.

### Limitations

While this study provides valuable insights into culture recovery in low-cost and crowded hospitals or diagnostic settings, it also has some limitations. The sample size of 66 individuals and 198 specimens was modest due to funding constraints that limited the ability to include a large cohort for robust statistical analysis. Recruiting fresh and untreated cases proved challenging, as many patients in developing countries with limited resources often begin treatment independently, influenced by the opinions of previously infected individuals or by quacks. The application of over-the-counter steroid antifungal creams before visiting the clinic may help reduce fungal growth and impact culture outcomes. Additionally, it is based on a single-center approach, which may limit its generalizability due to local hospital conditions and patient demographics. Expanding the study was not feasible due to resource constraints. Furthermore, as the primary objective of this study was to compare three different sampling approaches, the outcomes were measured utilizing only culture-based approaches which have its inherent limitations. We acknowledge that molecular confirmation of dermatophyte genetic material would provide additional reliability; future studies will incorporate such molecular validation to strengthen and extend these findings. Despite all these challenges, we believe the study rigorously evaluated sampling method performance, and the findings provide practical and reliable insights into improving dermatophytes culture under real-world, low-resource conditions.

## Ethics statements

Since this study involves direct human interaction, ethical approval was obtained from the Noakhali Science and Technology Ethics Committee, approval number: NSTU/SCI/EC/2022/91(A). Additionally, signed informed consent was obtained and documented for each patient enrolled in the study.

## CRediT authorship contribution statement

**Conceptualization**: Sabuj Baran Dhar (SBD), Firoz Ahmed (FA); **Data curation**: Mahmudul Hasan (MH), Ezaz Mahmud Sabit (EMS), Shinjon Ahmed (SA), Shariful Islam Sobuz (SIS), and Md. Abdullah Al Safi (AAS); **Formal analysis**: MH, SA, SIS and EMS; **Investigation**: Toslim Mahmud (TM), FA, and SBD; **Methodology and Writing -original draft**: MH, EMS, SA, SIS, and AAS; **Resources:** FA and SBD; **Software:** EMS; **Supervision**: TM, FA, SBD; **Validation:** EMS, TM, FA, and SBD; **Visualization:** MH; **Writing - review & editing:** MH, EMS, SIS, TM, FA, and SBD.

All authors have read and agreed to the submitted/published version of the manuscript.

## Declaration of competing interest

The authors declare that they have no known competing financial interests or personal relationships that could have appeared to influence the work reported in this paper.

## Data Availability

All data documented herein were supplied in supplementary files, and no data were utilized in any other research referenced in this publication.

## Appendix Supplementary materials

Image, application 1
